# Fungal Infections Complicating COVID-19: With the Rain Comes the Spores

**DOI:** 10.3390/jof6040279

**Published:** 2020-11-11

**Authors:** Alida Fe Talento, Martin Hoenigl

**Affiliations:** 1 Department of Microbiology, Children’s Health Ireland at Temple St., D01 YC67 Dublin, Ireland; 2 Division of Infectious Diseases and Global Health, University of California San Diego, San Diego, CA 92093, USA; 3 Clinical and Translational Fungal-Working Group, University of California San Diego, San Diego, CA 92093, USA; 4 Section of Infectious Diseases and Tropical Medicine, Medical University of Graz, 8036 Graz, Austria

Within the last 12 months, coronavirus disease 2019 (COVID-19) caused by the severe acute respiratory syndrome coronavirus-2 (SARS-CoV-2) spread globally to pandemic proportions. Although the majority of cases have asymptomatic or mild infections, a significant proportion progress to severe pneumonia and acute respiratory distress syndrome requiring critical care. Opportunistic infections following severe respiratory viral infections have been recognized since the 1918 influenza pandemic. Among critically ill patients with COVID-19, particularly secondary fungal infections caused by *Aspergillus* and *Candida* spp. are increasingly described. We, therefore, hosted a Special Issue focusing on fungal infections complicating COVID-19 and are delighted that a total of seven high quality papers were published within this issue. COVID-19-associated pulmonary aspergillosis (CAPA) has been reviewed in detail by Arastehfar et al., where authors have also shed light on the immunopathogenesis of CAPA, which is believed to occur due to a defective immune response in patients with severe COVID-19 leading to a hyperimmune state and dysfunctional T-lymphocytes infections [1]. The release of danger-associated molecular patterns during severe COVID-19 may contribute to pulmonary epithelial damage; collateral effects of host recognition pathways required for the activation of antiviral immunity may, paradoxically, contribute to a highly permissive inflammatory environment that favours the development of pulmonary mould infections [1]. CAPA has been shown to be associated with increased mortality that can only be lowered by early initiation of antifungal treatment [2,3]; thus, early diagnosis is essential. Mohamed et al. suggest screening patients with severe COVID-19 in intensive care who remain unwell using a combination of fungal biomarkers which include culture and galactomannan of deep respiratory samples, serum galactomannan and 1-3 beta-d-glucan, and molecular assays as well as computerised tomography [4]. Gangneux et al. demonstrated that molecular assays to detect Aspergillus DNA from blood and respiratory samples resulted in higher sensitivity when compared to culture based methods which may aid in the early diagnosis of CAPA [5]. Future studies should evaluate the role of point-of-care diagnostics for the diagnosis of CAPA, such as the Aspergillus Lateral Flow Device assay, which has shown promise for diagnosing pulmonary aspergillosis in the critical care setting [6].

Importantly, there are also reports of yeast infections in critically ill patients with COVID-19. While Arastehfar et al. point out that—in contrast to CAPA—there is no immunological predisposition, *Candida* blood stream infections may occur in patients with classical clinical risk factors including long-term ICU stays, indwelling vascular devices, and receipt of antibiotics and corticosteroids [7]. In another report in this Special Issue, two patients developed *Saccharomyces* blood stream infection after receipt of probiotics supplementation which contained the same strain of this yeast while in critical care [8], highlighting the risk of fungal translocation in these severely ill patients.

Lastly, but equally important, is the early initiation of appropriate antifungal therapy when secondary fungal infections are suspected. The global emergence of antifungal resistance in the two major fungal pathogens has made treatment more challenging given that there are only a few classes of systemic antifungal agents. Meijer et al. report on the first published case of CAPA due to a triazole-resistant *A. fumigatus* [9], while Posteraro et al. presents a case of a pan-echinocandin resistant *C. glabrata* bloodstream infection [10], with both cases leading to a fatal outcome. These cases underline the importance of performing antifungal susceptibility testing and antifungal stewardship.

As the global pandemic continues, we cannot overemphasise the need for a low threshold to screen for fungal infections for early diagnosis and allow appropriate antifungal therapy. Again, we express our sincere thanks to the authors and reviewers for their contribution to the literature on this very important topic, despite their busy schedules taking care of these patients with COVID-19.
